# Renormalization-group approach to quantum Fisher information in an XY model with staggered Dzyaloshinskii-Moriya interaction

**DOI:** 10.1038/srep19359

**Published:** 2016-01-19

**Authors:** X. M. Liu, W. W. Cheng, J. -M. Liu

**Affiliations:** 1 Laboratory of Solid State Microstructure, Nanjing University, Nanjing 210093, China; 2 Institute of Mathematical and Physical Sciences, Jiangsu University of Science and Technology, Zhenjiang 212003, China; 3 Institute of Signal Processing &Transmission, Nanjing University of Posts and Telecommunication, Nanjing 210093, China

## Abstract

We investigate the quantum Fisher information and quantum phase transitions of an XY spin chain with staggered Dzyaloshinskii-Moriya interaction using the quantum renormalization-group method. The quantum Fisher information, its first-derivatives, and the finite-size scaling behaviors are rigorously calculated respectively. The singularity of the derivatives at the phase transition point as a function of lattice size is carefully discussed and it is revealed that the scaling exponent for quantum Fisher information at the critical point can be used to describe the correlation length of this model, addressing the substantial role of staggered Dzyaloshinskii-Moriya interaction in modulating quantum phase transitions.

Quantum Fisher information (QFI)^1^,^2^, as a fundamental notion of quantum metrology^3^,^4^, plays a significant role in quantum detection and quantum estimation since it provides a bound to the accuracy of quantum estimation. The QFI is an extension of the Fisher information (FI) in the quantum regime that was originally introduced by Fisher^5^ and was defined by the Cramér-Rao bound^6^,^7^. The FI concept, arising from the statistics, quantifies the estimation precision of parameters and gives the optimal rate at which neighboring states can be distinguished by measurement. For a quantum system, the quantum Cramér-Rao inequality is proposed^8^,^9^,^10^,^11^ in which the QFI^1^,^2^ is set up. From the respect of metrology, the QFI is derived by maximizing the FI over all possible positive operator valued measurements (POVM). In the last decades, the QFI has been extensively investigated^12^,^13^,^14^,^15^,^16^,^17^,^18^,^19^,^20^,^21^,^22^,^23^, and the highly concerned issue is possible scheme to acquire a high estimation precision^12^,^13^,^14^,^15^,^16^,^17^,^18^,^19^,^20^. Along this line, researchers have developed various approaches, including utilizing the properties of input states such as coherence, entanglement, and spin squeezing to enhance the QFI since the bigger QFI the higher precision. On the other hand, for a practical system, both the de-coherence and the dissipation are unavoidable. This effect leads to substantial efforts in developing various strategies to obtain an optimal quantum measurement in such noisy systems^21^,^22^,^23^. In recent years, the problem of QFI correlation and its signature at quantum phase transitions have been receiving substantial attention. In fact, earlier work^24^ did prove the capability of the QFI in detecting the quantum criticality of the environment and this quantity does carry sufficient information on a lot of quantum phase transitions. Subsequently, we tested and then approved the QFI as a signature to characterize the localization transition of several one-dimensional quantum models^25^. Additionally, the scaling behaviors of QFI near the critical point of the XXZ spin chain model with the Dzyaloshinskii-Moriya (DM) interaction were discussed^26^, further demonstrating the QFI as a highly favorable measure of quantum information in a broad of quantum spin systems.

Apart from these functionalities of the QFI mentioned above, more essential issue is the characterization of the quantum phase transitions by the QFI. In particular, the quantum phase transitions in strongly correlated electron systems have been of long-standing and core ingredients in condensed matter physics. Basically, quantum phase transitions deal with zero-temperature phase transitions where quantum fluctuations play the dominant role. In such ground states, non-analytic behaviors of some physical quantities near the critical point will be identified often. In fact, no matter from the aspect of quantum information or quantum phase transition in condensed matters, one-dimensional spin models constitute the basis for understanding the physics and thus are highly valuable^27^,^28^. In details, spin systems such as the XY model, XXZ model and so on, have been used to investigate the quantum entanglement^29^,^30^,^31^,^32^,^33^,^34^, quantum discord^35^,^36^,^37^, and others, while the problem of quantum phase transitions is also addressed. Knowledge on these spin models allows peering into the ground states and low-energy excitation in more complicated quantum systems.

It should be mentioned that quantum phase transitions and associated magnetic properties would find more practical significance if the considered spin models account for more realistic exchange interactions which are comparable in energy and momentum scales with quantum fluctuations. For example, spin models including an antisymmetric super-exchange interaction such as the DM interaction^38^,^39^ which arises from the spin-orbit coupling become particularly attractive, while strong spin-orbit coupling has been suggested to be the core ingredient of physics for topological quantum systems, giving rise to more interesting quantum transitions. Nevertheless, it should be noted that the non-analytic behavior of order parameters in the vicinity of critical points for a quantum spin system is hardly detected unless sophisticated formulation scheme is applied. In this aspect, quantum renormalization-group (RG) method^40^,^41^ has been demonstrated to be valuable and convenient tool to rigorously treat the quantum phase transitions, and highly appreciated examples^42^,^43^,^44^,^45^ dealing with the non-analytic behaviors in the vicinity of critical points for quite a few systems are available. These progresses allow a possibility to utilize the quantum RG method for the QFI description of quantum phase transitions. In refs^42^,^43^,^44^,^45^, the physical quantities which have been investigated involve concurrence, entanglement, and discord. They are all quantum correlation measures from the quantum mutual information while QFI from the quantum estimation theory is a more intrinsic and ubiquitous quantity by comparison. Therefore, in this work, we combine the scheme of quantum RG and QFI to investigate the non-analytic behaviors of the quantum phase transition for the XY model with staggered DM interaction which is rather difficult to address analytically due to the introduction of DM interaction. It will be revealed that the derived QFI derivatives characterize very well the quantum critical point, and in particular the critical exponent of scaling behavior can measure the correlation length properly, enabling the QFI measure of quantum phase transitions well accessible.

## Results

### Quantum Fisher information

Firstly, we outline the QFI *F* for a quantum system. For a general phase estimation scenario, a quantum state evolves under the unitary transformation and can be expressed as *ρ*_*θ*_ = *e*^*−iAθ*^·*ρ* ·*e*^*iAθ*^, where *θ* is the parameter to be estimated and operator *A* is a generator. Correspondingly, the accuracy of estimating *θ* is limited by the quantum Cramér-Rao inequality^1^,^2^:


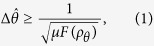


where *μ* is the number of experiments and *F*(*ρ*_*θ*_) is the so-called QFI. From another point of view, the QFI can be interpreted as the information on parameters encoded in the quantum state. Similarly, for an observable *O* on a system Hilbert space to be estimated, the QFI, i.e. the information involved in *ρ* with respect to observable *O*, can be given^1^,^8^,^9^,^46^:


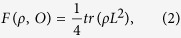


where *L* is the symmetric logarithmic derivative determined by the following equality:


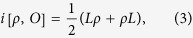


where [*ρ*, *O*] denotes the commutator between *ρ* and *O*. If *ρ* is a pure state, the QFI can be expressed as *F*(*ρ, O*) = *tr*(*ρO*^2^) − [*tr*(*ρO*)]^2^. In the case of mixed state, the calculation of QFI becomes more complicated and will be shown in details below.

Generally, a mixed state *ρ* can described as


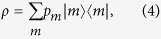


where |*m*〉 and *p*_*m*_ are respectively the eigenvectors and eigenvalues of the *m*-th components of state *ρ*. Substituting eqs (3) and (4) into eq. (2) allows the computation of the QFI^1^,^46^,^47^:


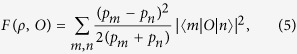


where *m* and *n* mark respectively the eigen-parameters (|*m*〉 and *p*_*m*_) and (|*n*〉 and *p*_*n*_).

For simplified calculation, we take a two-component composite system as an example and discuss the QFI calculation in details. Suppose that *ρ*_*ij*_ is any bipartite state, while {*A*_*u*_} and {*B*_*u*_} are respectively arbitrary local orthonormal observable bases of two subsystems in *ρ*_*ij*_. In this case, the QFI, encoded in this two-component system with respect to observables, can be evaluated as^46^


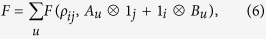


which is also called the global information of *ρ*_*ij*_.

On the other hand, it was proved that the QFI *F* in eq. (6) holds a fixed value^46^, regardless of the choice of local orthonormal bases. It means that *F* is an intrinsic quantity of the composite system. In this work, we apply the above framework of calculation to one-dimensional spin-1/2 XY chain which has been treated extensively. Without losing generality, we focus on two lattice sites of the spin chain as a composite system. In general, for a two-qubit system, the local orthonormal observables {*A*_*u*_} and {*B*_*u*_} can be taken as:


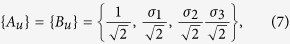


where *σ*_*i*_(*i* = 1, 2 and 3) are the Pauli matrices. Consequently, so long as *ρ*_*ij*_ is given, the QFI *F* can be calculated according to eqs (5, 6, 7).

### Renormalization of the Hamiltonian

Here, we apply the quantum RG method to the XY model with staggered DM interaction. It is known that the main objective of the RG method is to eliminate the less important degrees of freedom via a recursive procedure until a more tractable situation is reached. Following the Kadanoff’s block method, the Hamiltonian can be decomposed into the block Hamiltonian and interacting (inter-block) Hamiltonian. Each block is treated independently to obtain low-lying states and then build the projector onto the low-energy sub-space which can be called the effective Hilbert space. Subsequently, the inter-block interaction is projected onto this renormalized space. In this way, we can obtain an effective Hamiltonian which has structural similarity to the original Hamiltonian. Similar to ref. 26, we adopt the notion of “renormalization of QFI”, and study the quantum transitions of the XY spin chain with staggered DM interaction, discussing the non-analytic behavior of the QFI and the scaling behavior close to the critical point by evaluating the derivatives of the QFI.

It is well known that the Hamiltonian of a spin-1/2 XY model with staggered DM interaction along the *z* direction on a periodic chain of *L* sites can be described as^48^


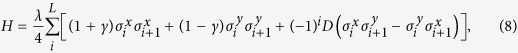


where *λ* is the exchange interaction strength, *γ* is the anisotropy parameter, and *D* is the strength of DM interaction in the *z* direction. The 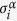 (*α* = *x*, *y*, *z*) are the Pauli matrices at site *i*. It should be mentioned that for the initial Hamiltonian eq. (8) the analysis shows that the effective Hamiltonian does not have a similar structure to the original. Therefore we implement the π rotation around the *x*-axis for even sites and leave all odd sites unchanged^49^. The transformed Hamiltonian can be written as


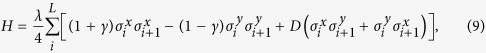


which allows the implication of the quantum RG method and the renormalization of coupling constants.

Correspondingly, the Hamiltonian of the spin chain is divided into two parts: the block Hamiltonian (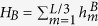) and inter-block Hamiltonian (*H*_*BB*_)^48^. Each block 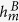 is composed of three sites and can be accurately diagonalized. The degenerate ground states of the block Hamiltonian are given as follows


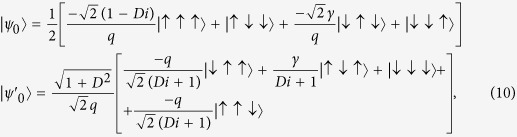


where 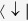 and  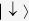 are the eigenstates of *σ*^*z*^ and *q* = (1 + *D*^2^ + *γ*^2^)^1/2^. The effective Hamiltonian of the renormalized spin chain at the *n*-th RG step can be cast into the form


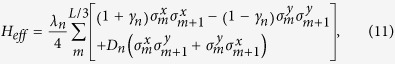


where subscript *n* labels the renormalized parameters upon the *n*-th RG step. The iterative relationships between these parameters at the *n*-th RG step and (*n* + 1)-th RG step are:


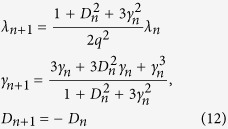


In the following, we will calculate the QFI, taking one of the degenerate ground states 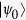 as example. The block density matrix is defined by


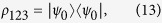


If one considers another ground state 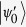, the result is the same. By tracing the density matrix *ρ*_*123*_ on the middle site 2 of the block, the reduced density matrix between sites 1 and 3 can be written as


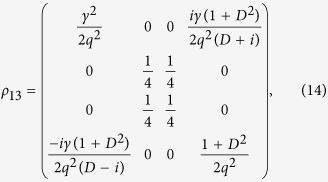


According to the above description, we can derive the QFI of quantum state *ρ*_*13*_:


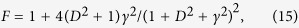


The calculations show that the QFI depends on the anisotropy parameter *γ* and the DM interaction *D*. In the following section, we will discuss the evolution behavior of QFI as a function of (*γ*, *D*) in details and analyze the critical properties of the spin chain utilizing the derivatives of QFI and its scaling behavior.

### The analysis of QFI

**Figure 1:**
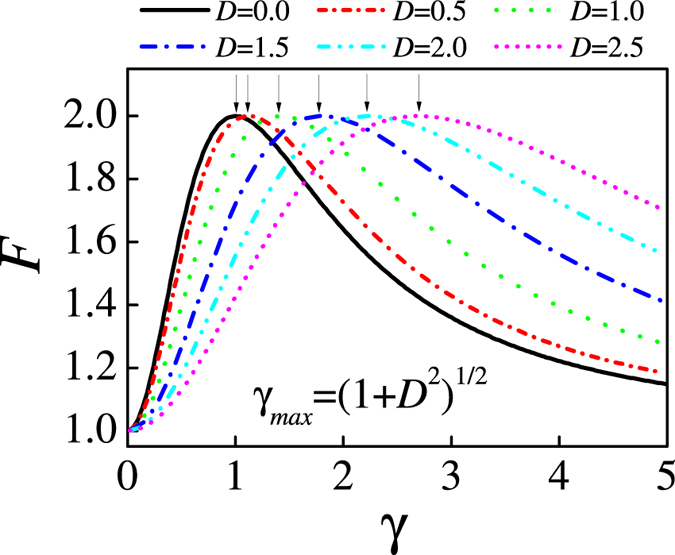
The evolution of QFI with respect to the anisotropic parameter as DM interaction *D* = 0, 0.5, 1.0, 1.5, 2.0, and 2.5 for a three-site model.

**Figure 2:**
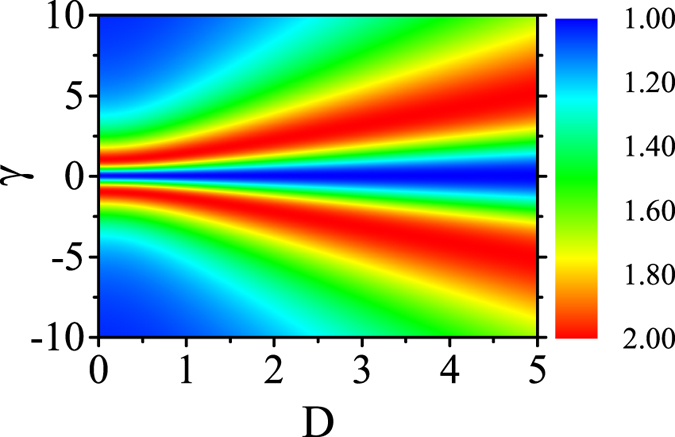
The evolution of QFI in the parameter space (*D*, *γ*) for a three-site model.

In Fig. 1 are shown the QFI data for the three-site model with respect to parameter *γ* at different *D* values. The QFI has fixed value *F* ≡ 1.0 at *γ* = 0 regardless of *D*, and this property can be easily seen from eq. (15). In this case, the system is actually the XX model, implying no phase transitions. Given each value of *D*, the *F*(*γ*) is a single-peaked function. The peak heights remain identical but the peak location shifts rightwards with increasing *D*. One can easily determine from eq. (12) that the peak location *γ* = *γ*_*max*_ = √(1 + *D*^2^) as defined by the analytic calculation of *dF/dγ* = 0. This behavior reflects the competition between the anisotropy effect and DM interaction in the XY model with staggered DM interaction. In Fig. 2 we show the distribution of QFI in the parameter space (*D*, *γ*). From this graph, one sees clearly how the QFI evoles with respect to the anisotropic parameter *γ* given a DM interaction.

**Figure 3:**
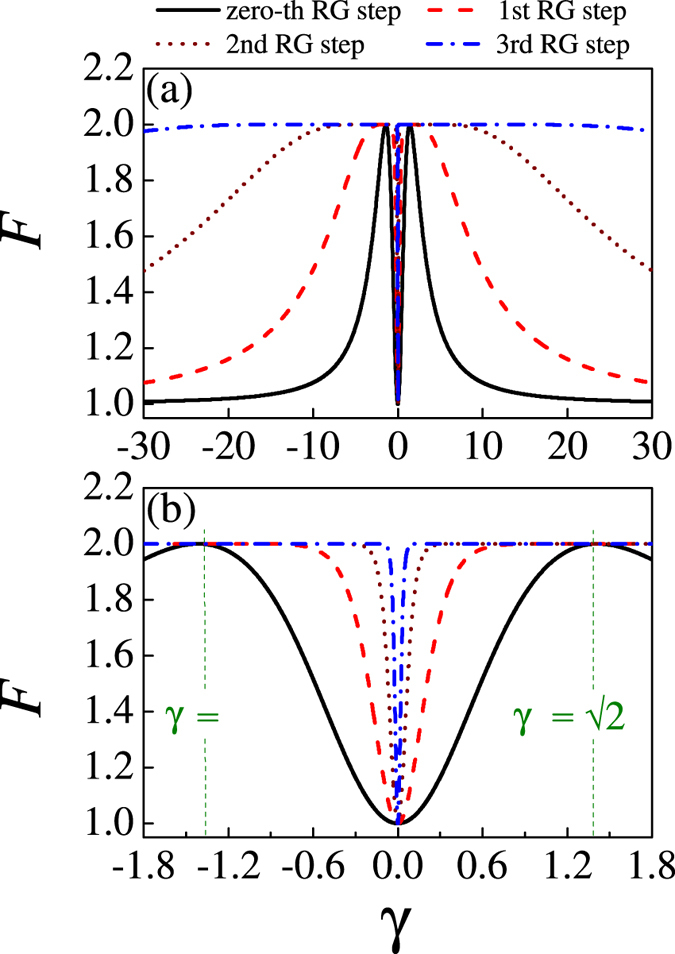
The evolution of QFI with respect to the anisotropic parameter at a fixed value of DM interaction *D* = 1 in terms of RG iterations. (**a**) −30 ≤ *γ*  ≤ 30, (**b**) −1.8 ≤  *γ*  ≤ 1.8.

The quantum RG procedure enables the global property of the XY spin model to be tracked upon the proceeding RG iteration. In the other words, completing the *n*-th RG step allows a lattice with 3^*n* + 1^ sites to be effectively represented by a three-site block. In this case, the QFI measures the information of the effective degree of freedom, i.e. two parts of the system. To illustrate this effect more clearly, we plot in Fig. 3(a) the *F*(*γ*) curves obtained upon several RG iterations (*n* = 0 to *n* = 3). The graph shows the evolution of QFI with *γ* at quantum RG iterations, given *D* = 1.0. The enlarged details around *γ* ~ 0 are presented in Fig. 3(b). Several distinct features can be found with the increasing chain size upon the RG iteration. First, the QFI has the minimal point *F* ≡ 1.0 at *γ* = 0 no matter how many RG iterations, implying that the system is always in the spin-fluid phase at *γ* = 0. Second, the QFI tends to the minimal *F* ≡ 1.0 as *γ* →±∞. This implies that the system is also in the spin-fluid phase no matter how many RG iterations are done. It is reasonable since the original model reduces to the isotropic XX model at *γ* = 0 and *γ *→ ±∞. Third, for the zero-th RG step (*n* = 0), the QFI reaches the maximal *F* ≡ 2.0 at *γ *≡ ±√2. The system is in the Néel phase. Upon the increasing RG iterations, i.e. increasing lattice size, this Néel phase can be stabilized over the continuously broadened ranges around *γ* = ±√2, as shown clearly in Fig. 3(b). Finally, all the *F*(*γ*) curves obtained at different *n* (RG iterations) coincide at points *γ* = 0 and *γ* = ±√2, suggesting that these points are all the fixed points. In fact, if one rewrites eq. (15) into:


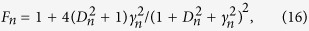


where *F*_*n*_ is the QFI at the *n*-th RG step, it is clear that *F*_*n*_ ≡ 1.0 at *γ*_*n*_ = 0 and *F*_*n*_ ≡ 2.0 at *γ*_*n*_ = ±√2. However, it is noted that the point *γ* *=* 0 is an unstable fixed point, i.e. a possible critical transition point to be discussed below, while the points *γ* = ±√2 are two stable fixed points. The RG procedure enables the unstable fixed point *γ* = 0 more precisely definable, as the consequence of the RG treatment.

**Figure 4:**
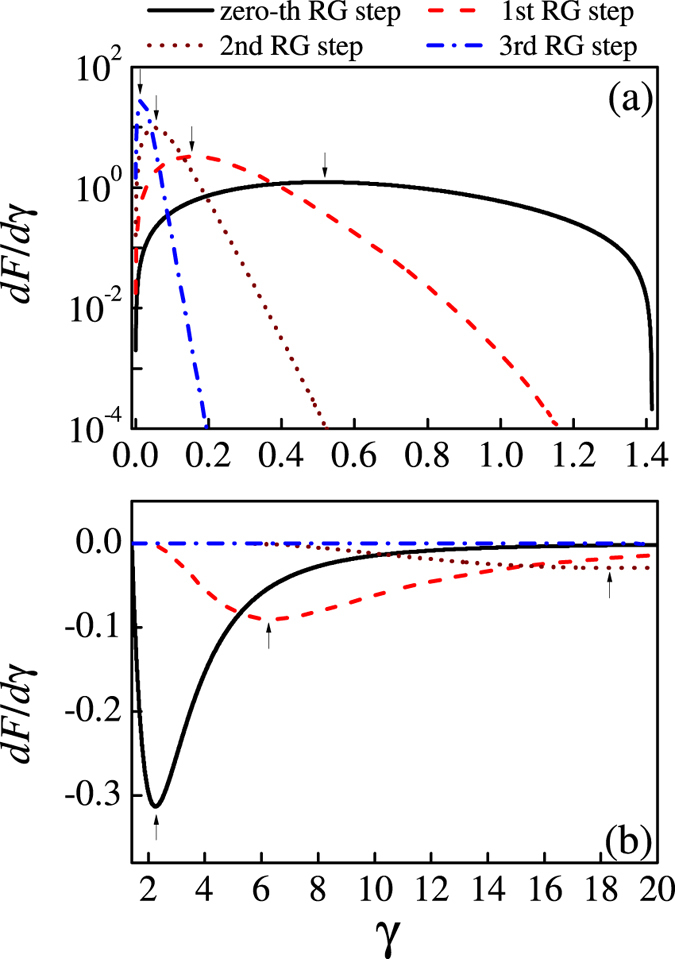
First derivative of QFI *dF/dγ* at a fixed value of DM interaction *D* = 1 as the size of the system increases. (**a**) 0 ≤ *γ* ≤ √2, (**b**) √2 ≤ *γ* ≤ 20.

**Figure 5:**
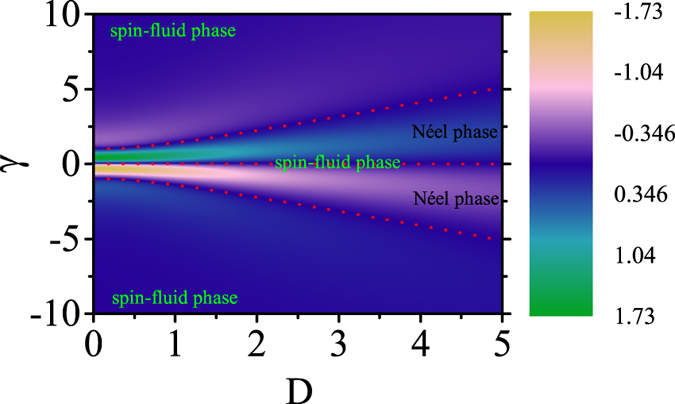
First derivative of QFI *dF/dγ* in the parameter space (*D*, *γ*) for a three-site model. The transition lines (the dotted lines) divide the whole space into several parts presenting the different phase.

To further uncover the system behaviors at the possible critical point *γ* = 0, one can investigate another parameter of order so that the critical phase transition can be discussed. Here we look at the first-order derivative of QFI, *dF*(*γ*)/*dγ*, as a measure of the second-order phase transition. In Fig. 4(a) are plotted the *dF/dγ* curves obtained at different *n* (0, 1, 2, 3) within 0 ≤ *γ *≤ √2 and the curves as *γ* ≥ √2 are shown in Fig. 4(b). The results as *γ* < 0 are mirror-symmetric with those as *γ* > 0. It is seen from Fig. 4(a) that the maximal of *dF/dγ* calculated from the zero-th RG step appears at *γ* ~ 0.5, the increasing RG iteration shifts the maximal of *dF/dγ* towards *γ* ~ 0.0 and eventually the singularity at *γ* = 0 will be reached when the RG iteration increases till *n* → ∞. It is thus demonstrated the point *γ* = 0 is indeed a critical phase transition point. On the other hand, as shown in Fig. 4(b), the minimal of *dF/dγ* at *n* = 0 appears at *γ* ~ 2.2 and it will shift toward the infinity at *n* → ∞ at which *dF/dγ* = 0 over the whole *γ* range. Therefore, the RG iterated results on *dF/dγ* are consistent with the iterated results on *F*, indicating that the point *γ* = 0 rather than *γ* = ±√2 is the critical phase transition point. Here, we can state derivatives of QFI not only give the scaling behavior at the critical point but also the scaling properties at the stable fixed points. For other DM interaction values, we can similarly obtain the evolution of QFI with respect to parameter *γ*. From the singular behavior of *dF/dγ*, we can derive the same critical point *γ* = 0. Meanwhile, the stable fixed points can be obtained from the infinity of the derivative *dF/dγ*. The calculated results are presented in Fig. 5. The position of the color mutation represents the transition point. By detailed analysis, the corresponding phases are labelled in the figure.

**Figure 6:**
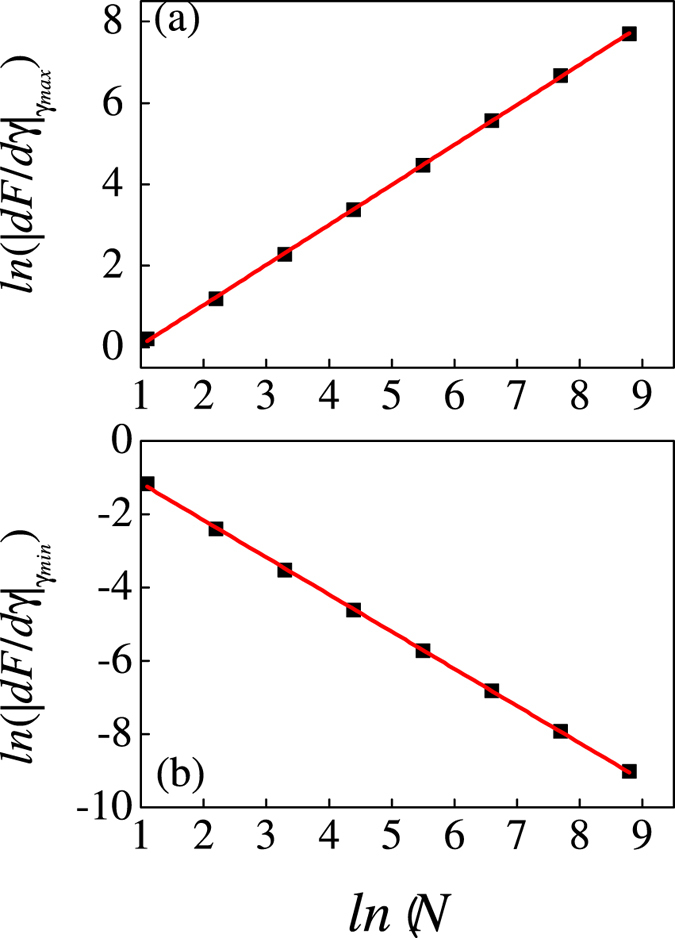
The scaling behavior of |*dF/dγ*|_*γ*max_ (**a**) and |*dF/dγ*|_*γ*min_ (**b**) with respect to the system size (|*dF/dγ*|_*γ*max_ is the absolute value of maximum in Fig. 4(a) and |*dF/dγ*|_*γ*min_ is the absolute value of minimum in Fig. 4(b)).

In Fig. 6(a), we have shown the scaling behavior of *dF/dγ* versus *N* in the vicinity of the phase transition point. Here the position of the maximum of *dF/dγ* in Fig. 4(a) is described as *γ*_*max*_. Consequently, the *ln*|*dF/dγ*|_*γmax*_ has a linear behavior versus *ln(N)* and the exponent for this behavior is |*dF/dγ*|_*γmax*_ *~* *N*^*0.99*^. In addition, when the iteration tends to infinity, there is also minimum for each plot (the position of the minimum of *dF/dγ* is *γ*_*min*_) which has been shown in Fig. 4(b). Different from the case with *γ*_*max*_, the minimum of derivatives decays down to zero when the system approaches the thermodynamic limit. These also indicate that there are the same properties for *γ* = 0 and *γ* = ±∞. Similarly, in Fig. 6(b), we have plotted the scaling behavior of *ln*|*dF/dγ*|_*γmin*_ versus *N*. The corresponding exponent is −1.0.

**Figure 7:**
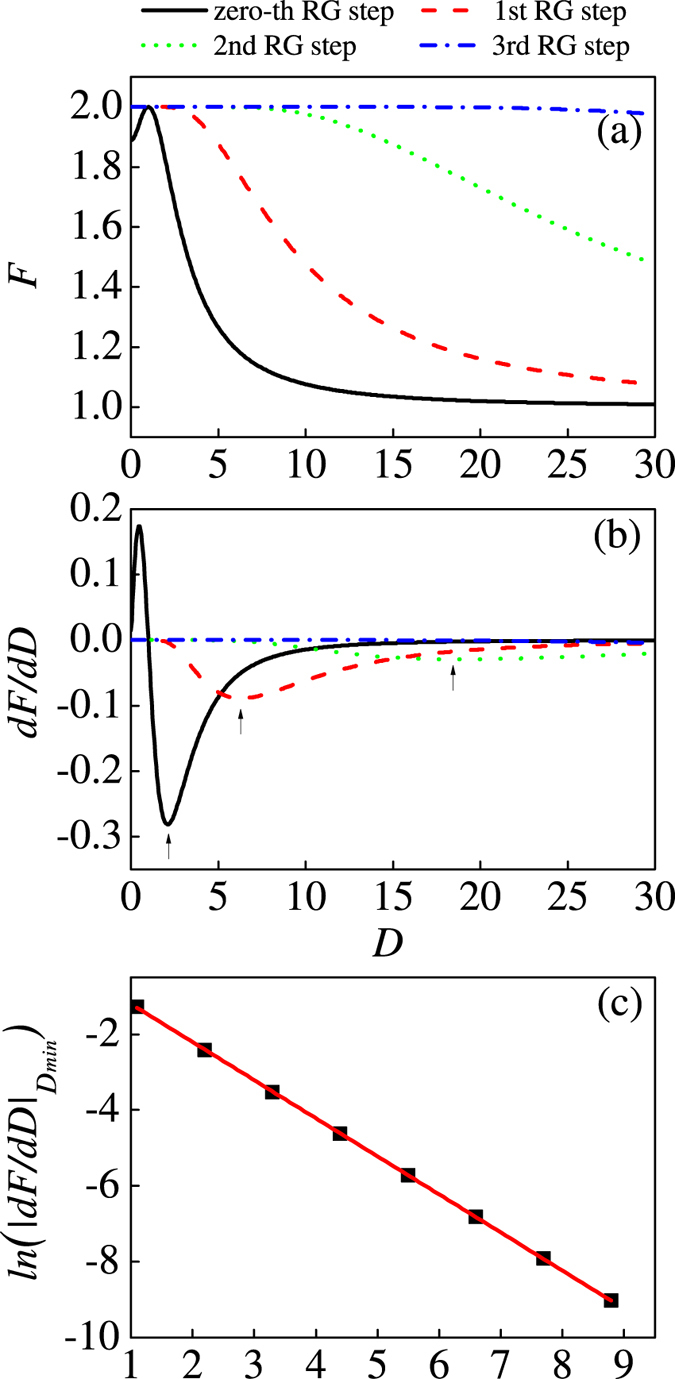
(**a**) The evolution of QFI with respect to the DM interaction at a fixed value of the anisotropic parameter *γ* = √2 in terms of RG iterations. (**b**) First derivative of QFI *dF/dD* at a fixed value of the anisotropic parameter *γ* = √2 as the size of the system increases. (**c**) The scaling behavior of |*dF/dγ*|_*Dmin*_ with respect to the lattice size (here |*dF/dγ*|_*Dmin*_ is the absolute value of minimum for different iterations in Fig. 7(b)).

On the other hand, in Fig. 7(a) we also have plotted QFI versus *D* at a fixed value of anisotropic parameter *γ* = √2 for different iterations. It is found that QFI reduces with increasing of DM interaction, finally reaching the minimum value. This hints that at *γ* = √2, the variants of DM interaction cannot cause quantum phase transition. This can be confirmed by derivative of QFI versus *D* which has been shown in Fig. 7(b). As the number of RG iterations increases, the minimum of derivative *dF/dD* (the position of the minimum is *D*_*min*_) becomes bigger. When the system reaches infinity, |*dF/dD*|_*Dmin*_ tends to 0. Meanwhile, we have derived the scaling behavior of |*dF/dD*|_*Dmin*_ versus *N* in Fig. 7(c). The linear relation can be expressed as |*dF/dD*|_*Dmin*_ ~ *N*^−1.0^. It should be mentioned that this exponent is associated with the correlation length exponent close to the critical point, just the reciprocal of the correlation exponent.

All the above results and analysis demonstrate that QFI, combined with the RG method can be a good description of critical behavior of the XY model with staggered DM interaction.

## Discussions

In this paper, we have applied the quantum RG method to calculate quantum Fisher information of the anisotropic XY spin chain with staggered DM interaction. First, by the three-site model, we have found the competition between the anisotropic effect and DM interaction. Second, the evolution of QFI in RG iterations exhibits a quantum phase transition point *γ *= 0 and three stable fixed point *γ *= ±√2 and *γ* = ∞. This can be further affirmed by the first-derivative of QFI with respect to the anisotropic parameter and DM interaction. For the critical point, the derivative becomes singular with increasing of the system size while for the points *γ *= ±√2, the derivative has no singularity, presenting the tendency of slow variation. At these points, the corresponding scaling behaviors versus the lattice size are obtained. The results demonstrate that in the thermodynamic limit, the nonanalytic behavior of QFI is correlated with the divergence of the correlation length at the critical point. Thus, we can obtain the critical exponent of the system. From the above results, we can see that the quantum Fisher information, assisted by the quantum RG method, is promising and expected to be applicable to more other spin systems.

## Additional Information

**How to cite this article**: Liu, X. M. *et al.* Renormalization-group approach to quantum Fisher information in an XY model with staggered Dzyaloshinskii-Moriya interaction. *Sci. Rep.* **6**, 19359; doi: 10.1038/srep19359 (2016).
